# Combination Anthelmintic/Antioxidant Activity Against *Schistosoma Mansoni*

**DOI:** 10.3390/biom9020054

**Published:** 2019-02-05

**Authors:** Maria João Gouveia, Paul J. Brindley, Gabriel Rinaldi, Fátima Gärtner, José Manuel Correia da Costa, Nuno Vale

**Affiliations:** 1 Center for the Study in Animal Science, University of Porto, (CECA/ICETA), Rua de D. Manuel II, Apt 55142, 4051-401 Porto, Portugal; jose.costa@insa.min-saude.pt; 2 Department of Molecular Pathology and Immunology, Institute of Biomedical Sciences Abel Salazar (ICBAS), University of Porto, Rua de Jorge Viterbo Ferreira 228, 4050-313 Porto, Portugal; fgartner@ipatimup.pt (F.G.); nuno.vale@ff.up.pt (N.V.); 3 Department of Drug Sciences, Laboratory of Pharmacology, Faculty of Pharmacy, University of Porto, Rua de Jorge Viterbo Ferreira 228, 4050-313 Porto, Portugal; 4 Department of Microbiology, Immunology & Tropical Medicine, Research Center for Neglected Diseases of Poverty, School of Medicine & Health Sciences, George Washington University, Washington, DC 20037, USA; pbrindley@email.gwu.edu (P.J.B.); gr10@sanger.ac.uk (G.R.); 5 Institute of Molecular Pathology and Immunology of the University of Porto (IPATIMUP), Rua Júlio Amaral de Carvalho 45, 4200-135 Porto, Portugal; 6 University of Porto, i3S, Instituto de Investigação e Inovação em Saúde, Rua Alfredo Allen 208, 4200-135 Porto, Portugal; 7 Department of Infectious Diseases, INSA-National Institute of Health Dr. Ricardo Jorge, Rua Alexandre Herculano 321, 4000-055 Porto, Portugal

**Keywords:** *Schistosoma mansoni*, antioxidant biomolecules, anthelmintic drug, anticancer drug, drug repurposing, combination therapy, newly transformed schistosomula

## Abstract

Schistosomiasis is a major neglected tropical disease. Treatment for schistosomiasis with praziquantel (PZQ), which is effective against the parasite, by itself is not capable to counteract infection-associated disease lesions including hepatic fibrosis. There is a pressing need for novel therapies. Due to their biological properties, antioxidant biomolecules might be useful in treating and reverting associated pathological sequelae. Here, we investigated a novel therapy approach based on a combination of anthelmintic drugs with antioxidant biomolecules. We used a host-parasite model involving *Bioamphalaria glabrata* and newly transformed schistosomula (NTS) of *Schistosoma mansoni*. For in vitro drug screening assays, was selected several antioxidants and evaluated not only antischistosomal activity but also ability to enhance activity of the anthelmintic drugs praziquantel (PZQ) and artesunate (AS). The morphological alterations induced by compounds alone/combined were assessed on daily basis using an inverted and automated microscope to quantify NTS viability by a fluorometric-based method. The findings indicated that not only do some antioxidants improve antischistosomal activity of the two anthelmintics, but they exhibit activity per se, leading to high mortality of NTS post-exposure. The combination index (CI) of PZQ + Mel (CI = 0.80), PZQ + Resv (CI = 0.74), AS + Resv (CI = 0.34), AS + NAC (CI = 0.89), VDT + Flav (CI = 1.03) and VDT + Resv (CI = 1.06) reveal that they display moderate to strong synergism. The combination of compounds with discrete mechanisms of action might provide a valuable adjunct to contribution for treatment of schistosomiasis-associated disease.

## 1. Introduction

Since the 1970s, chemotherapy against schistosomiasis has relied on praziquantel (PZQ) which is considered the drug of choice for infection [1,2]. Despite PZQ efficacy against all forms of schistosome species presenting mild and transient side effects, it has major drawbacks as its poor efficacy against juvenile parasites and pharmacokinetic profile (e.g., extensive first-pass metabolism) [1,2]. In addition, massive and exclusive reliance on a single drug raises legitimate concern about schistosome PZQ-resistance. In fact, studies have reported field and experimental isolates that exhibit significantly reduced susceptibility, which might be a foreshadowing for emergence of resistance [3,4]. Moreover, PZQ-based treatment alone cannot reverse pathological sequelae of infection as periportal fibrosis for intestinal schistosomiasis and bladder deformity and hydronephrosis for urogenital schistosomiasis [5,6]. Taken together these considerations, there is a pressing need to search for additional therapeutics. Drug repurposing is an efficient approach to reduce time and cost of drug research and development [7,8], and/or combination of discrete biological active agents [9]. From our perspective, novel therapeutic approaches should focus not only on antischistosomal performance but also in amelioration of disease sequelae. During schistosomiasis, oxidative processes are triggered by liberation of reactive oxygen species (ROS) resulting from the immunological response and disturbance in cellular antioxidant homeostasis of affected organs [10,11]. We have suggested that reactive electrophilic compounds, e.g., estrogen-like metabolites of parasite origin initiate the carcinogenesis process associated with the chronic infection with *Schistosoma haematobium*. These metabolites are capable of interacting with host DNA leading to formation of DNA-adducts (releasing ROS) triggering a cascade of events that may lead to squamous cell carcinoma (SCC) of bladder associated to infection [12,13].

Due to the physiological properties of antioxidants, which are considered pharmacologically safe agents with minimal side effects [14,15] we speculated that combining these agents with anthelmintic drugs might offer enhanced antischistosomal action in combination with PZQ or other anti-worm drugs. Antioxidants have demonstrated antischistosomal activities [16], exhibiting differential performance among developmental stages of the parasite [17,18,19,20,21]. Moreover, the antioxidants resveratrol (Resv) and *N*-acetylcysteine (NAC) have demonstrable ability to prevent DNA damage [21] and block carcinogenesis [22,23,24,25].

Based on findings with drug repurposing and combinations of multiple biological active agent strategies, we report a novel therapeutic approach that combined anthelmintic drugs and anticancer with antioxidants biomolecules. We hypothesize that combinations of these active agents would reveal synergies that could be translated into enhanced antischistosomal activity. We demonstrated that not only do some antioxidants alone exhibit antischistosomal activity inducing severe morphological alterations and death of parasite but that they improve activity of established drugs including PZQ and artesunate (AS). AS is an antimalarial that unlike PZQ is effective against the juvenile forms of the schistosome [7,16]. In addition, we have also assessed the combination of anticancer drugs combined with antioxidant biomolecules. It has been demonstrated that kinase inhibitors as imatinib (IMT) interfere with essential development steps in the biology of schistosomes and causes degenerative changes in the gonads and gastrodermis [26,27,28]. Therefore, we included these agents here and evaluated antischistosomal activity in the presence of antioxidant biomolecules. The findings presented below reinforced the notion that combining biological agents with discrete modes of action might provide a valuable approach for treatment of schistosomiasis.

## 2. Materials and Methods

### 2.1. Chemicals and Culture Media

Praziquantel (PZQ), 4-phenyl-1,2,5-oxadiazole-3-carbonile-2-oxide (OXA), *N*-acetylcysteine (NAC), flavona (Flav), flubendazole (FBZ), and propidium iodide (PI) were purchased from Sigma-Aldrich (Lisboa, Portugal), and resveratrol (Resv) from Santa Cruz Biotechnology (Heidelberg, Germany), artesunate (AS), vandetanib (VDT), curcumin (Curc), kaempferol (kaempf), and melatonin (Mel) from Cayman Chemical (Ann Arbor, MI, USA) and the dipeptide H-L-tryptophan-L-serine-OH (H-Trp-Ser-OH, DiPept) from Bachem (Bubendorf, Switzerland). The culture media M199, Hank’s Balanced Salt Solution (HBSS) and supplements as HEPES (4-(2-hydroxyethyl)-1-piperazineethanesulfonic acid) (1M), penicillin (10,000 U/mL)/streptomycin (10 mg/mL), and amphotericin B were purchases from Sigma-Aldrich and heat inactivated fetal bovine serum (iFBS) from Lonza (Basel, Switzerland). For in vitro assays, stock solutions of (2–4 mg/mL) were freshly prepared in 100% dimethylsulfoxide (DMSO) (Sigma-Aldrich) and stored at 4 °C. 

### 2.2. Parasites 

Experimentally-infected *Bioamphalaria glabrata* snails maintained in water were exposed to artificial light during 2–3 h to shed cercariae of *Schistosoma mansoni* (isolate of Brazilian origin) maintained on Department of Infectious Diseases of National Institute of Health Dr. Ricardo Jorge (Porto). The number of cercariae in suspension was estimated and mechanically transformed into newly transformed schistosomula (NTS) as described [29,30] but with some modifications, as follows. The cercarial suspension was placed on ice to for 60 min to decrease motility of the larvae. Subsequently, the cercariae were pelleted by centrifugation and then resuspended in HBSS, 2% amphotericin B, vigorously stirred and slightly vortexed to induce tail loss tail. To recover the NTS, cold HBSS was added to the suspension and chilled on ice for 15 min. The supernatant now rich in tails was decanted and the pellet was resuspended in 7 mL cold HBSS. These steps were repeated at least twice. The conversion rate was calculated by counting the number of cercariae before transformation and the total NTS after enrichment. The NTS suspension was adjusted to a concentration of 50–100 NTS per 100 µL culture medium M199, 10% iFBS, 1% penicillin/streptomycin and dispensed into wells of a 96 well flat bottom plate (Nunclon, Roskilde, Denmark). Before addition of the compounds, the suspension was incubated at 37 °C, 5% CO_2_ for 24 h to ensure complete conversion of cercariae to NTS. 

### 2.3. In Vitro Drug Sensitivity Assay in NTS

The antischistosomal activity of anthelmintic, anticancer drugs and antioxidant biomolecules, either alone or combined (at ratio 1:1), was evaluated at concentration of 100 µM since it more suitable to test sublethal doses of natural product derivatives as antioxidants [31,32]. In combination, each drug and antioxidant were evaluated at 100 µM. To evaluate the effectiveness of novel therapeutic approach the 96 well flat bottom plates were prepared as follow: 1) 150 µL of pre heated (37 °C) supplemented M199 was placed in each well; two) dilutions of compounds (alone or combined) were added to achieve final concentration of 100 µM, and, three) NTS suspension was added to achieving final volume of 250 µL/well. The NTS containing the highest DMSO concentration (2% *v*/*v*) served as vehicle control. Initially, viability and morphological alteration at 1, 17, 24, and 48 h post-exposure to compounds were assessed using by inverted microscopy under bright field at 10–40× magnification (Nikon Phase Contrast 2, LDW 0.52, Tokyo, Japan) coupled with a camera (Canon PowerShot A360, Tokyo, Japan). To assess the phenotypic changes, we used a semi-quantitative viability scale as described below (Table 1).

At 72 h post-exposure, an automated microscope LionHeart FX (BioTek, Winooski, VT, USA) incorporated with Gen5 3.0 software to process and analyze the data, was used in color bright field or fluorescence channel Texas Red (586 nm) to assessed NTS viability. In order to quantify the viability, a fluorophore (a solution of 0.5 mg/mL of PI) was added to the wells and incubated for 15 min at 37 °C [34]. Subsequently, the plates were read on the LionHeart FX microscope system using a fluorescence channel. The PI incorporate membrane-compromised cells staining with red fluorescence. Therefore, the parasites stained and with no movement for 90 s were considered to be dead [35]. The performance of each combination was characterized using a combination index, as described [36], and calculated as follows [37]:

Combination index (CI) = [(% NTS dead due to anthelmintic drug) + (% NTS dead due to antioxidant)]/(% NTS dead in the presence of the drug combination)(1)

where the classification used has been described by other investigators [38], where CI > 0.1 very strong synergism, CI: 0.1–0.3 strong synergism, CI: 0.3–0.7 synergism, CI: 0.7–0.85 moderate synergism, CI: 0.85–0.9 slight synergism, CI: 0.9–1.1 nearly additive and CI > 1.1 antagonist. Each concentration of anthelmintic and anticancer drug, antioxidant either alone or combined was tested in duplicate, and the assays were performed at least twice. 

## 3. Results

### 3.1. Anticancer Drugs Demonstrated Interesting Antischistosomal Activity in Comparison to Classic Anthelmintic Drugs

The NTS incubated with culture media only (without DMSO and compounds) exhibited normal viability with slight morphological alterations such as increased granularity and reduced motility. At completion of the incubation/assays, the NTS remained active with 92.8 ± 6.6% viable and score 2 according criteria of viability for phenotypic alterations (Table 1). The NTS incubated with DMSO (2%) presented similar viability, 92.4 ± 10.0% and morphological alterations. DMSO at 2% did not induce significant phenotypic alterations in NTS in comparison to NTS incubated with medium only. In Table 2 presents the mean and standard deviation (SD) percentage of NTS viability obtained during the different experiments on controls, vehicle control (DMSO), PZQ, and AS.

We evaluated two classes of drugs with discrete antischistosomal activity: the anthelmintics AS, PZQ and FBZ and the anticancer drugs IMT, and VDT at 100 µM. Viability of NTS following exposure to these compounds is presented in Table 3. Among the anthelmintics, FBZ was more active than AS and PZQ. Regarding to the anticancer agent’s VDT was most potent against NTS leading to death of most NTS; only 29.4% remained alive (Table 3).

**Figure 1:**
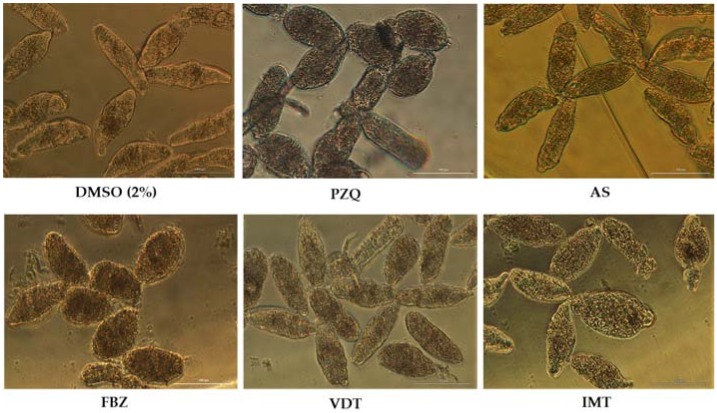
Representative micrographs of newly transformed schistosomula (NTS) at 72 h of exposure to the anthelmintics praziquantel (PZQ), artesunate (AS), and flubendazole (FBZ) and anticancer drugs vandetanib (VDT), and imatinib (IMT) at 100 µM. Compared to controls, NTS incubated with drugs showed severe morphological alterations, and the morphological changes differed among the drugs. Scale bar, 100 µm.

PZQ at 100 µM induced several and severe morphological alterations following 72 h post-exposure. At one hour, the NTS were hyperactive with increased granularity and altered body shape. During the assay, the movement of NTS decreased and vacuoles and blebbing were induced (Figure 1). The NTS incubated with PZQ showed a mean phenotypic viability score of 1 or 0.5. Whereas the scoring system is semi-quantitative only, it does indicate that the NTS were severe altered although not dead. Indeed by 72 h some NTS remained alive and were actively moving. Moreover, the evaluation of viability using PI demonstrated that although they were severely altered, many NTS remained unstained and viable 50.0 ± 23.9%. The standard deviation was high due to inconsistent percentage of viability through assays with PZQ varying between 7.8 up to 71.0% (Table 1). It does not appear that drug lost activity since it continued to induce morphological alterations in NTS consistent with previous reports [35].

The NTS incubated with antimalarial and antischistosomal drug, AS at 100 µM showed inconsistent results (Table 2). In some assays, after 72 h the NTS presented dark body due severe granularity and no activity. Moreover, the tegumental membrane seemed to be disrupted. In these assays, all NTS were stained with PI and therefore were considered dead (0% viability). Curiously, in other assays performed under the same conditions, NTS did not show significant morphological alterations (Figure 1). Both the viability score or the viability assessed with PI were similar to controls. We hypothesized that stability of AS in solution decreased and consequently the drug might be degraded which could be translated in loss of antischistosomal activity, as reported by others [39]. Intriguingly, in a follow-up assay, the AS killed all the NTS following 72 h post-exposure; the mean and standard deviations reflected these uneven results (87.5 ± 49.5)%. 

FBZ achieved better antischistosomal activity than PZQ and AS. Following 72 h post-exposure, NTS treated with FBZ had rounded up, were blebbed, exhibited minimal activity, severe granularity and altered (wrinkled) tegumental membrane (Figure 1). Viability as assessed with PI at 72 h revealed that mean of viability was 7.4%. The morphological alterations induced by FBZ were similar, although more marked, to PZQ. At least, both anthelmintic drugs induced the rounded phenotype. Although not all the NTS were killed by FBZ and by PZQ, these anthelmintics induced severe and irreversible morphological alterations. Therefore, we could speculate that the development from schistosomulum to adult parasite would be compromised. 

Concerning the anticancer agents, VDT and IMT showed potent to moderate activity against NTS at 100 µM. During evaluation of VDT at 100 µM, the morphological alterations were more pronounced at 72 h exposure, displayed altered shape, increased granularity and lack of movement at lower magnification (Figure 1). Assay of viability with PI revealed that VDT induced death in most (~70%) of the NTS, while those remaining alive showed minimal or no movement. VDT at 25 µM showed reduced antischistosomal activity; at 72 h exposure, VDT induces slight morphological alterations similar to those observed to controls, and percentage viability assessed with PI was not different from the controls (not shown). IMT showed moderate activity. By 72 h, morphological alterations in NTS included granularity (Figure 1). The majority of NTS remain alive and unstained by PI (72.3% viable) and viability score of 2 (Table 1 and Table 3). NTS incubated with IMT was more active and less altered than VDT.

### 3.2. Antioxidants Alone Showed Potent Antischistosomal Activity

Eight antioxidants were studied: Resv, Flav, Curc, Mel, Kaempf, OXA, H-L-Trp-L-Ser-OH (DiPept), and NAC. All were evaluated at 100 µM alone and combined (see below) with the drugs listed earlier. Findings for antioxidants alone shown in Table 4. 

**Figure 2:**
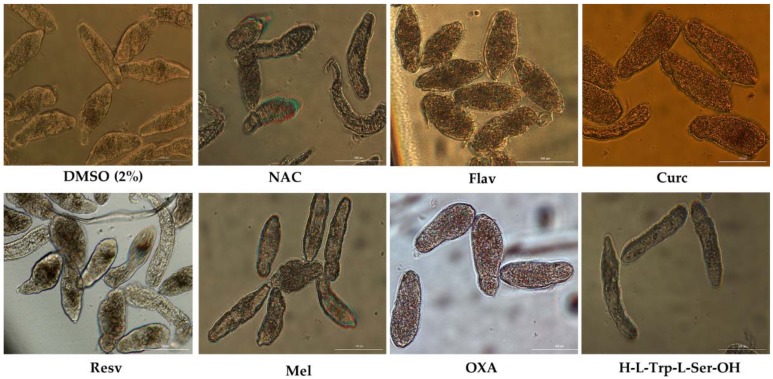
Representative micrographs of NTS at 72 h of incubation in antioxidants at 100 µM. Notable differences were evident among controls and NTS incubated with 4-phenyl-1,2,5-oxadiazole-3-carbonile-2-oxide (OXA), curcumin (Curc), and flavone (Flav), which induced severe granularity and reduced activity. In contrast to OXA and Curc, Flav did not kill all the NTS. Resveratrol (Resv) also induced moderate morphological alterations but did not kill all NTS. *N*-acetylcysteina (NAC), melatonin (Mel) and dipeptide (DiPept) did not induce significant morphological alterations. Scale bar, 100 µm.

Two antioxidants, Curc and OXA, demonstrated a striking antischistosomal activity, killing all NTS before the end of experiment. The morphological alterations induced by Curc and OXA were dissimilar, suggesting different modes of action. At one-hour exposure to Curc, the schistosomula showed severe morphological changes including severe granularity, altered shape. Curc killed all NTS by 1 h. NTS incubated with OXA at the same period did not present significant morphological alterations, but they had no movement. Following 17 h exposure, NTS incubated with OXA showed increases granularity, blebbing, and lack of movement. Figure 2 shows NTS incubated with the eight antioxidants at 72 h. At the end of experiment, viability of NTS was assessed and confirmed by staining with PI.

Among the other antioxidants, Resv and Flav exhibited moderate antischistosomal activity, and significant morphological alterations (Figure 2). Following 72 h post-exposure, Flav had caused severe granularity and minimal activity in NTS, corresponding to viability score of 1 (Table 1). Despite the morphological alterations, 59.3% NTS remained alive. Resv also induced morphological alterations into NTS, although not as pronounced as by Flav. Most NTS incubated with Resv had severe granularity, minimal activity, alteration of shape. Some exhibited only mild granularity and no alteration of shape. These NTS had a score viability of 1.5 and 43.1% viability when assessed with PI (Table 4).

The antioxidants, Mel, DiPept and NAC failed to cause significant antischistosomal effects. At 72 h, NTS had a percentage of viability similar to controls (~90%) (Table 4). The NTS remained viable during the experiment without significant morphological alterations and only marginal increase of granularity and decrease of activity, with a viability score of 2.5. Notably, and by contrast, following 72 h exposure to DiPept, NTS were more active than controls suggesting that this antioxidant might enhance maintenance of NTS in vitro. Evaluating the antischistosomal activity of Kaemp was not feasible because a precipitate formed upon addition of the stock to wells containing NTS in culture medium, evidently the result of interaction of Kaempf with proteins in culture medium [40]. The NTS exhibited morphological alterations, but we could not ascribed the effects to Kaempf due to the obvious perturbation of the culture medium. 

### 3.3. Antischistosomal Activity of Combination of Drugs and Antioxidants Show That Antioxidants Might Enhance Activity of Drugs

**Figure 3:**
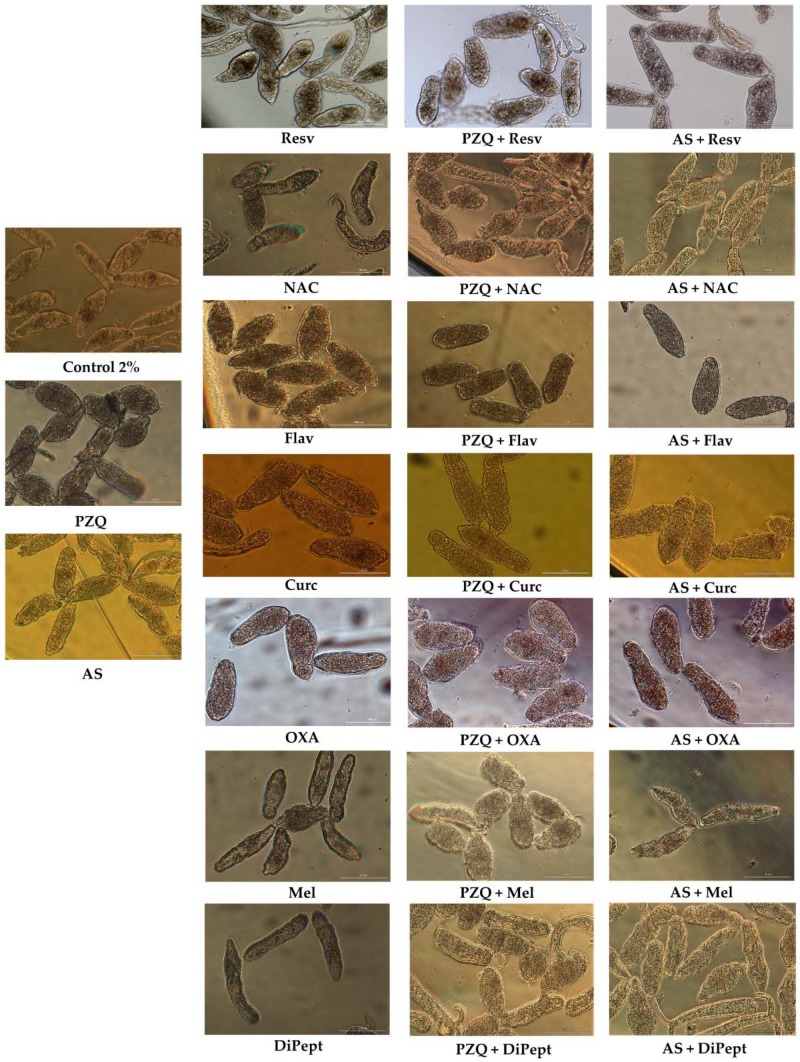
Representative micrographs of NTS at 72 h after exposure to combinations of praziquantel (PZQ) and artesunate (AS) with several antioxidants at 100 μM, and 1:1 constant ratio; scale bar, 100 μm.

Among the combinations of PZQ with antioxidants, PZQ + Curc and PZQ + OXA achieved better results leading to death of all NTS before 72 h of exposure in a similar fashion to observed with these antioxidants alone (Table 5). Therefore, the potent antischistosomal activity observed in combinations was related to the antischistosomal activity of antioxidants itself. The morphological alterations observed with these combinations were more similar to those induced by antioxidants alone rather PZQ (Figure 3). Intriguingly, the combination index demonstrated that PZQ combined with Curc or OXA exhibited slightly antagonism (Table 5). 

The antioxidants Mel and Resv enhanced antischistosomal activity of PZQ. The combination of these antioxidants, i.e., PZQ + Mel and PZQ + Resv leads to a significant decrease of NTS viability in comparison to either compound alone. In fact, the combination index demonstrated that they are slightly synergistic. The larvae incubated with combinations had similar morphological alterations induced by PZQ alone (Figure 3); nonetheless, they were less active and viability was lower than for the compounds alone (Table 5).

The NTS treated with combinations of PZQ with Flav, DiPept, and NAC achieved better percentage of viability than compounds alone. Comparing the viability of PZQ (7.8%) and Flav (59.3%) alone with PZQ + Flav it was possible to observe a significant increase (72.9%). Apparently, Flav suppresses the antischistosomal activity of PZQ acting as antagonist as expresses by CI (Table 5). Despite the percentage viability in PZQ + Flav, NTS were severely damaged and minimum activity was detected (Figure 3), yet, they remained alive—they did not stain with PI (not shown). This suggest that compounds did not damage the tegumental membrane of the NTS. Comparing morphological alterations of NTS incubated with compounds alone or combined, some differences were apparent among them. The morphology of NTS incubated with PZQ + Flav was different from those incubated with compounds alone (Figure 3). The body shape was marginally different from Flav being more similar to PZQ. All NTS had severe granularity and minimal activity, in accord with the viability score of 1 (Table 1).

The antioxidants DiPept or NAC combined with PZQ slightly improved viability of NTS in comparison to drug alone. Taken into consideration the CI, we classified these combinations as additive (Table 5). The morphological alterations observed in NTS treated with these combinations were similar to those observed with PZQ (Figure 3), but the viability score was 1 rather 0.5 attributed to PZQ alone. Also, the percentage of viability assessed with PI revealed that in combinations were slightly higher than PZQ alone (Table 5). As noted above, this may relate to the fact that these antioxidants might enhance viability of NTS in vitro.

In general, combinations of AS with antioxidants were more active than the compounds alone. In like fashion to findings described above, exposure for one or 17 h to combinations AS + Curc and AS + OXA caused the death of NTS, respectively. The effects were related with activity of antioxidants alone rather than to enhancement of antischistosomal activity of AS. These two combinations were classified as nearly additive (Table 6). The morphological alterations observed in NTS were similar to those described for antioxidants alone (Figure 3), supporting the notion that antischistosomal activity was due to antioxidants alone and not to AS.

The AS + Resv combination resulted in death of all NTS by 72 h following drug exposure whereas AS alone was not lethal to all the NTS. Comparing the morphological alterations induced by AS and Resv alone and combined, e.g. AS + Resv, it was clear that they were more pronounced in NTS treated with the combination. It is noteworthy that the membrane of NTS was compromised and disrupted (Figure 3). Assessing viability by PI staining revealed that all NTS treated with the combination were stained (0% viability) in contrast to incubation with AS and Resv alone (Table 6). Accordingly, Resv enhanced antischistosomal activity of AS, as previously observed [30].

In contrast to PZQ + Flav, the combination of AS + Flav reduced viability almost to half of viability observed in AS alone. This reduction was less pronounced when comparing viability of Flav and AS + Flav (Table 6). It is interesting to note that the same antioxidant behaved in a different manner when combined with different drugs. The morphological alterations observed in NTS treated with AS + Flav were similar to those induced by Flav alone; however, NTS shape was more oval (Figure 3). Nonetheless, both NTS incubated with Flav and AS + Flav had minimum, almost undetectable activity and their viability score was 0.5. By contrast, with AS, movement was apparent as were slight morphological alterations consistent to viability score of 2. In fact, the viability assessed by PI confirms these observations, as we can observe on Table 6, Flav had a lower viability compared to AS alone. Similarly, in combination, the viability was also lower than compounds alone. This combination was classified as nearly additive (Table 6).

The combination of NAC or DiPept with AS resulted in decreased viability compared to the compounds alone; however, the reduction was not pronounced (~10%) and morphology of NTS were similar to controls. In general, NTS did not present significant alterations besides slight increase of granularity and decrease of activity. Regarding AS + DiPept, NTS were less active than those incubated with DiPept alone. This combination was classified as additive while AS + NAC was slightly synergistic (Table 6). Interestingly, NTS incubated alone or in combinations presented same phenotypic viability score of 2. 

The combination of AS + Mel was considered as antagonist, in contrast to PZQ + Mel. In this case, NTS incubated with AS + Mel presented slightly better viability compared to the compounds alone (Table 6). Similar to combinations with NAC or DiPept, NTS incubated with AS + Mel did not present any significant morphological alterations, only a slight increase of granularity and decrease of activity but remained viable with a similar phenotypic viability score (2.5) and percentage (~90%) to those of the controls and compounds alone (Figure 3; Table 6).

FBZ was evaluated in combination with the antioxidants, DiPept, Resv, and Flav. These antioxidants were selected based on the results obtained in the combinations with PZQ and AS. Table 7 presents the viability (%) of NTS after exposure to combinations with FBZ and the combination index.

**Figure 4:**
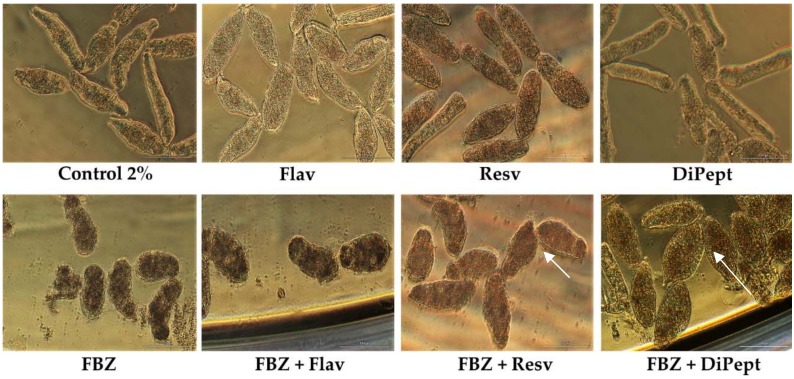
Representatives micrographs of NTS incubated with combinations of FBZ with Flav, Resv, and DiPept at 100 µM and constant ratio 1:1 at 72 h post-exposure. Notably, the morphologic alterations in combinations were more similar to drug alone rather antioxidant, specifically FBZ + Flav. In combinations FBZ + Resv and FBZ + DiPept NTS were more swollen and altered in shape (white arrows). Scale bar, 100 µm.

Notably, combining FBZ and DiPept improved survival of the NTS compared to FBZ alone (Table 7). This is intriguing, but similar to observations for the combination PZQ + DiPept, although less pronounced. However, NTS incubated with FBZ + DiPept following exposure for 72 h did not show detectable activity. Nonetheless, most were still alive since they did not stain with PI (viability, 95.7%). The morphological alterations induced by combination were similar to NTS incubated with FBZ alone: a rounded shape with tegumental blebbing (Figure 4). The outcome was similar to that for PZQ and Flav alone; severe alterations but many remained viable. Also, DiPept might be important for the maintenance of the tegument and its membrane and to prevent the entry of PI. According to the CI, this combination was highly antagonistic (Table 7).

When FBZ was combined with Resv or Flav, NTS were dead by 48 h after exposure in both cases, as confirmed by PI staining. This outcome likely was due solely to drug since all NTS incubated with FBZ alone also died (Table 7). Nonetheless, the morphological alterations induced by combinations FBZ + Resv and FBZ + Flav were different. The NTS incubated with FBZ + Flav displayed an oval form and darker body, similar to FBZ alone.

On the other hand, NTS expose to FBZ + Resv were more elongated with severe granularity which is more consistent with morphological alterations induced by Resv (Figure 4). Probably, Resv reverses the morphological alterations induced by FBZ but did not affect its activity given that all the NTS subsequently died. Curiously, both combinations, FBZ + Resv and FBZ + Flav, were classified as marginally antagonistic (Table 7).

We also evaluated antischistosomal activity against NTS of anticancer drugs, VDT, and IMT combined with several antioxidants. The results achieved by anticancer drugs in combination with antioxidants are listed in Table 8.

**Figure 5:**
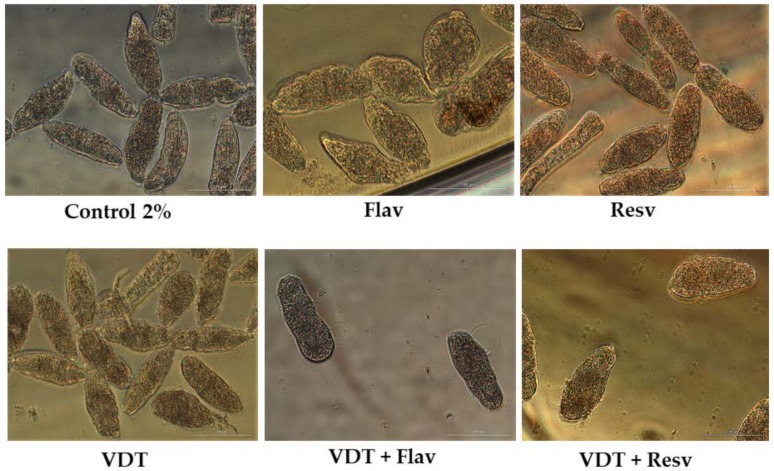
Representative micrographs of NTS at 72 h of incubation in VDT, Flav and Resv either alone or combined at 100 µM and 1:1 constant ratio; scale bar, 100 µm.

The anticancer drug, VDT was evaluated combined with Flav and Resv. As we mention above the drug itself and antioxidants (Flav and Resv) had antischistosomal activity against NTS. Interestingly, the combination of VDT with Resv and Flav resulted in a significant increase of antischistosomal activity leading to death of NTS. The percentage of viability achieved by these combinations was near 0% (Table 8). In fact, it is clear that the combination of these antioxidants with VDT reveal additive effect as confirm by CI depict on Table 3. The morphology of NTS incubated with combinations was different than on compounds alone and control (Figure 5). In case of VDT + Flav, NTS were neither similar to Flav alone or VDT while in VDT + Resv they were more similar to Resv alone although more oval shape rather vemiform.

**Figure 6:**
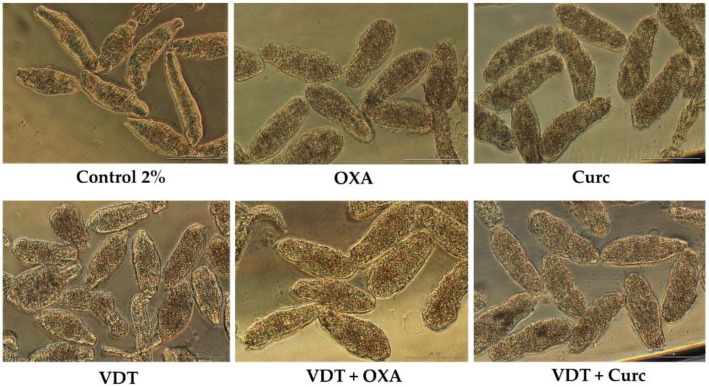
Representative micrographs of NTS incubated with combinations of VDT, Flav, and Resv alone and in combination at 25 µM and 1:1 constant ratio at 72 h post exposure. Scale bar: 100 µm.

We also evaluated the combination of VDT with Curc or OXA at constant ratio of 1:1 but at a lower concentration, 25 µM. In both combinations, VDT + Curc and VDT + OXA, all the NTS were dead at the end of experiment due to antischistosomal activity of the antioxidants (see above) and not due to anticancer drug. The combinations of VDT + Curc and VDT + OXA killed all NTS following 1 h exposure to the antioxidants alone. This is related to antischistosomal activity of anioxidants that alone also killed all the NTS at the same time (above). In these combinations, NTS showed similar morphological alterations to those induced by antioxidant alone (Figure 6). 

**Figure 7:**
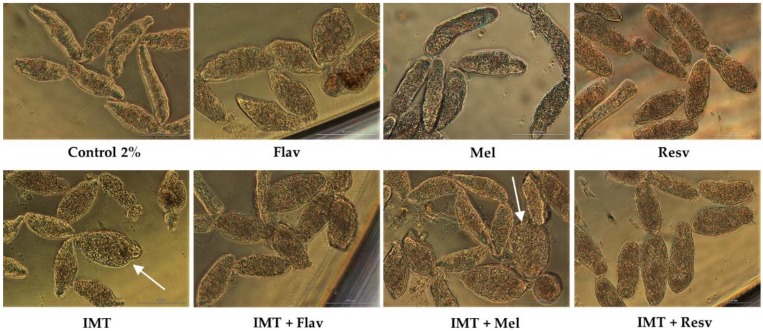
Micrographs of NTS at 72 h of exposure to IMT, Flav, and Resv, Mel alone or combined at a concentration of 100 µM and 1:1 constant ratio; x20; bright field; scale bar, 100 µm.

With IMT, we evaluated its combination at constant ratio 1:1 and 100 µM with Flav, Resv, and Mel. Following 72 h exposure, IMT combined with Flav and Resv did not enhance antischistosomal activity. NTS treated with combinations had similar viability to anticancer drugs or antioxidants alone (Table 8). Nonetheless, NTS treated with combinations induced morphological alterations that in the case of IMT + Flav were consistent with Flav alone although with more granulation. With IMT + Resv, NTS had severe granularity and altered, swollen body shape (Figure 7). The combination indexes for IMT + Flav and IMT + Resv suggested that they are slightly antagonist.

In contrast, the combination of IMT + Mel resulted in increase of percentage of viability (91.3%) in comparison to IMT alone (77.3%) and a slight decrease when compared to Mel alone (98.1%) (Table 8). Nonetheless, NTS showed morphological alterations consistent to those induced by IMT alone. Some of NTS were round and swollen (white arrow) while others remained elongated but different from Mel alone, whereas Mel did not revert the morphological alterations, it rescued NTS from death.

#### The Combination of Two Antioxidants (OXA + Curc) is Highly Potent Against NTS

**Figure 8:**
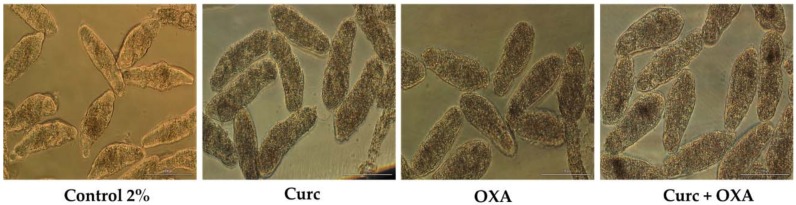
Representative micrographs of NTS at 72 h of exposure to Curc and OXA and combined, at 25 µM and 1:1 constant ratio; ×20; bright field; scale bar, 100 µm.

In order to understand whether Curc or OXA was the more potent antioxidant, we conducted an in vitro assay to evaluate the combinations against NTS at the lower concentration of 25 µM. However, even at this lower concentration we could not determine which one was more potent since both killed all the NTS by 17 h after exposure. Curcumin induced more morphological alterations and impaired movement earlier than OXA. At one hour, similar to 100 µM, NTS incubated with OXA was more similar to controls whereas NTS in Curc had already altered. In combination, all NTS were dead by 17 h following exposure. The morphological alterations of NTS in Curc + OXA were more similar to those induced by OXA than Curc (Figure 8). 

Nonetheless, the cooperative index for this combination was two, which indicated that these drugs were antagonists. This was unexpected and anomalous to what we had observed. In fact, all NTS were killed either by antioxidants alone but also by combination in a similar time of exposure. Note that dead of all NTS either incubated with antioxidants alone or in combination was confirmed through staining of PI.

## 4. Discussion

The current treatment for schistosomiasis based only on PZQ [2] cannot fully counteract the infection associated morbidity and sequelae including periportal fibrosis, esophageal varices, and, with *S. haematobium* infection, sandy patches of the lower female genital tract [41], and squamous cell carcinoma of the bladder [5,12,42]. In the present study, we propose a novel therapeutic strategy that combined principles of drug repurposing and the combination of different active agents. Drug repurposing is based on previous investigations and given that some drugs are already commercialized, novel antischistosomal drugs could quickly advance into clinical testing, diminishing the time and cost of new drug development [7]. Our aim is achieved synergistic and/or additive effect that not only eliminate the parasite but also ameliorate morbidity associated to infection. Here we evaluated the ability of biomolecule antioxidants to enhance antischistosomal activity of anthelmintic and anticancer drugs against the schistosomulum stage of *S. mansoni* in vitro. These biomolecules were investigated due to their biological properties and the possibility of antischistosomal activity per se [16]. 

We evaluate three anthelmintic, PZQ, AS, and FBZ known to be active against different developmental stages of the *Schistosoma* species and other parasites. Whereas PZQ is more effective against adult worms, AS is more active against larval stages [43]. The anthelmintic drug FBZ has broad spectrum action against tapeworm infections. In vivo studies (in mice) demonstrated its activity in reducing numbers of adult parasites of *S. mansoni* [44,45]. Here, FBZ was more potent than PZQ or AS against NTS. Therefore, FBZ not only is active against adult worms but also against the schistosomulum stage. Based on in vitro and in vivo data it seems that FBZ is active against several developmental stages of *S. mansoni*. This antischistosomal activity is of interest since it suggests that it might overcome one of the limitations of PZQ and even AS. The mechanism of action of FBZ encompasses its ability to specifically bind and interact with microtubules that are main components of the eukaryotic cytoskeleton [46]. Probably FBZ interacts with microtubule inhibiting the surface membrane maturation of NTS and consequently altered its shape and leading to death [47]. In regard to the mechanism of action of AS and PZQ, they remain uncertain, but AS action may be related to the presence of endoperoxide bridge that induces the production of ROS whereas PZQ might act through the calcium channels [2,43].

The three anthelmintic drugs induced different morphological alterations which is probably related to its different targets on NTS and to their discrete modes of action. Nonetheless, these morphological alterations are more similar between PZQ and FBZ compared to AS. Both drugs induce an oval shape worms (rather than vermiform) and severe granularity. However, reduction of viability was more pronounced with FBZ. Although PZQ and FBZ induces severe morphological alterations on NTS, they did not kill the NTS, consistent with other reports [48]. 

We also evaluated the anticancer drugs IMT and VDT which are kinase inhibitors. The importance of kinases in members of the family Schistosomidae has been reviewed extensively [49]. Schistosome kinases play pivotal roles for different physiological processes, including reproduction, which is closely associated with egg production and the pathology of schistosomiasis. The anticancer drugs alone had demonstrated moderate to potent activity against NTS. Most likely the antischistosomal activity of IMT and mostly VDT are linked to the fact that they might impair the kinases of NTS which are very important to their development [49]. In next phase, we will assess which schistosome kinases are possible affected by these anticancer drugs.

The use of antioxidants against schistosomiasis have been reviewed [16]. Here we selected several antioxidants and evaluated not only antischistosomal activity but also its ability to enhance antischistosomal activity of both anthelmintics and anticancer agents. Of the antioxidants evaluated, Curc and OXA were highly active, and quickly killed the schistosomula. These findings were consistent with others reports [50]. Curcumin has demonstrated antischistosomal activity in vitro against adult worms and also affects the development of eggs [51]. The mechanism by which Curc exerts in vitro antischistosomal effect against both larval and adult worms is uncertain. However, Curc has a direct action involving in parasite biochemical processes. One possible target is the ubiquitin-proteasome pathway [52]. Proteasome inhibitors reduce the number of lung stage schistosomula, the worm burden and consequently decrease the egg output in infected mice [50,51]. Therefore, it is reasonable to hypothesize that in vitro antischistosomal activity of Curc against NTS might be due to inhibition of the ubiquitin-proteasome pathway. Regarding OXA, antischistosomal activity against NTS might be related to its ability to inhibit thioredoxin glutathione reductase (TGR) [19]. This enzyme is essential for parasite survival and is biochemically distinct from host enzymes; also, the parasite redox system is dependent of TGR [53]. Inhibition of TGR activity would lead directly to the inactivation of both thioredoxin and glutathione-based defenses and the accumulation of ROS and RNS species [19]. The results obtained for these two antioxidants suggested that antischistosomal activity might be related to different modes of action. The combination of these two antioxidants at lower concentrations also translated into potent antischistosomal activity. The morphological alterations induced by combination of antioxidants were different in comparison to those induced by antioxidant alone. 

Flav and Resv demonstrated similar moderate antischistosomal activity against NTS. The results obtained for Resv were consistent to findings that we reported previously [30]. Comparing the morphological changes induced by the two antioxidants, they were more pronounced in NTS incubated with Flav. Resv might act on neuromotor activity based on its effects on motility, which in turn could degrade its ability to migrate and acquire nutrients [30]. It is worth emphasizing that in experimental schistosomiasis in murine model Resv ameliorates oxidative stress and organ dysfunctions [54]. Since Resv had moderate antischistosomal activity here, maybe the results obtained by Soliman et al. are not related, not only due to its biological properties but also its antischistosomal activity against parasites. The mechanism of action of Flav against NTS is unclear. Flav has shown a broad spectrum as an anticancer and antioxidant, among other attributes. Its anticancer effects are exerted through binding receptor estrogens [25]. This is interesting since *Schistosoma* spp. produce/excrete estrogen-like metabolites to host that triggers a cascade of events that culminate in cancer in the case of *S. haematobium* infection [12,13]. Maybe the effects of Flav in NTS are associated to these metabolites or its inhibition. It is unclear why and how these metabolites are acquired and their role in parasite. Some authors attribute broad spectrum activities of Flav to their capacity to modulate key cellular enzyme function [55]. Further studies are necessary to evaluate its antischistosomal activity against adult worms and the possible drug targets.

In contrast, Mel, DiPept and NAC did not exhibit significant antischistosomal activity. Interestingly, NTS incubated with these antioxidants were more active than controls. These compounds likely are necessary for NTS maintenance, at least in vitro. Other reports demonstrated Mel also did not present antischistosomal activity against adult worms [56]. Nevertheless, some studies reported that they might ameliorate morbidity associated to infection. For example, NAC and Mel in vivo studies ameliorate redox homeostasis by downregulating oxidative stress caused by infection reducing fibrotic area and granulomas [56,57]. Mel might be use in immunization program, also might has multiple direct and indirect antioxidant actions as ability to stimulate host antioxidant enzymes and mitochondrial oxidative phosphorylation [58]. This antioxidant might be responsible for the immunoprophylactic effect and could protect host against infection [16]. In the case of DiPept, its antischistosomal activity was evaluated for the first time. Despite, it did not demonstrate antischistosomal activity it might be useful in ameliorate of pathology. NTS incubated with DiPept were more active rather controls, an outcome that might be related to the fact that dipeptides are building blocks of proteins and thus, the acquisition of these small peptides is critical for protein anabolism necessary for larvae in vitro. Regarding Kaempf it was impossible evaluate its antischistosomal activity since this antioxidant interacts with proteins presented in culture media precipitating [39]. For evaluation of its activity it would be necessary to use fetal bovine serum free medium which might compromise NTS viability. 

The results obtained by combinations were interesting and demonstrated that even though some antioxidants did not display significant activity against NTS, that does not necessarily indicate that they were not able to enhance antischistosomal activity of drugs. Interestingly, this effect was more pronounced in the case of combinations with AS and anticancer VDT. In terms of combination index, combinations of AS or VDT with antioxidants achieved better results with most of them classified as additive or synergistic. Based on these results, it seems that AS and VDT present a better profile for the combination of different active agents preventing the nullification of the activity of one compound over the other.

Nonetheless, PZQ combined with Resv or Mel were also classified as synergistic. The synergism in antischistosomal activity could be a result from increased action against anthelmintic drug targets or by acting concomitantly on discrete targets [59]. By contrast, some combinations were classified as antagonists including PZQ + Flav, PZQ + OXA, PZQ + DiPept, FBZ + Resv, FBZ + Flav, IMT plus Flav or Resv and AS + Mel. Although they were classified as antagonistic it should be noted that it a slightly antagonist (CI between 1.75 and 1.29). In most of these cases, the percentage of viability for NTS incubated with combinations slightly increased in comparison to the compounds alone. Yet, it does not necessarily mean that combinations of drugs plus antioxidant annuls the effect of each other. In the case of combinations such as PZQ + Flav, AS + Mel, FBZ + DiPept, and IMT + Mel which exhibited CI values ranging 2–21, the percentage of viability in NTS incubated with combinations increased comparatively with compounds alone. We hypothesize that combinations of the compounds might inhibit antischistosomal activity of either the drug or the antioxidant. The same antioxidant incubated with different drugs displayed different combination indexes. The antimalarial AS combined with Mel presented a CI = 0.80 suggesting synergism. Interestingly, a combination of the same antioxidant with a different drug, such as IMT or Mel, did not enhance antischistosomal activity of drug. Indeed, the CI revealed that they acted as antagonists. In a similar fashion, DiPept combined with AS achieved an additive effect; however, in combination with FBZ and PZQ, DiPept acts as an antagonist (more pronounced in FBZ + DiPept). In this case, it seems that the antioxidant inhibits the antischistosomal activity of drug. As mentioned above maybe NTS requires DiPept for their maintenance in laboratory culture or that interaction of both compounds inhibits FBZ activity. These findings suggested that the activity of the antioxidant varies depending on the drug used for the combination. In general, morphological alterations were more pronounced in NTS treated with combinations rather compounds alone, even if it did not result in their death. Most likely, severely altered NTS could not growth and develop into adult worms. Accordingly, this strategy might be of value for elimination of schistosomes, at least, the larval stage of *S. mansoni*. Further studies, including in vivo studies using the rodent model of schistosomiasis are required for evaluation of its efficacy not only against adult worms but also in amelioration of morbidities associated with the infection.

## 5. Conclusions 

Not only did the antioxidants exhibit antischistosomal activity but several antioxidants enhanced the schistosomical activity of the anthelmintics drugs. The additive or synergistic effects achieved by combinations of antioxidant and anthelmintic might be related their different mode of action and/or different targets on NTS. Repurposing of drugs as FBZ or anticancer drugs (or indeed other classes) might be worthwhile since they were effective against the schistosomulum stage of *S. mansoni*. This approach might be considered for prophylaxis or for use in regions with intense re-infection levels since the combination might block or retard parasite infection and development. Investigation of effects can now proceed to confirm the synergies of these combinations reported here against adult forms, and in vivo in laboratory rodents and likewise in other schistosome species including *S. haematobium*. Due to the biological properties of antioxidants in prevention of DNA damage and blocking cancer initiation processes [21,22,23,24,25], antioxidants may also counteract carcinogenesis during infection with helminth parasites [12,13,60,61]. 

## Figures and Tables

**Table 1:** Semi-quantitative viability scale used to evaluate phenotypic changes of *Schistosoma mansoni* newly transformed schistosomula (NTS) induced by compounds alone and its combinations [8,33].

Viability Scale	Phenotypic Alteration
0	All worms dead; severe granularity
1	Minimal activity; severe morphological changes; granularity
2	Showed activity; first morphological change; granularity visible
3	Totally vital; normal activity; gross morphological changes not apparent

**Table 2:** Viability of NTS of *S. mansoni* (mean ± SD) at 72 h of exposure in vitro to praziquantel (PZQ), artesunate (AS), or DMSO (vehicle) at 100 µM.

% NTS Viability							
Control (w/o DMSO)	Mean ± SD	Vehicle Control (DMSO)	Mean ± SD	PZQ	Mean ± SD	AS	Mean ± SD
94.4	92.8 ± 6.6	69.4	92.4±10.0	34.8	50.0 ±23.9	0.0	87.5 ± 49.5
96.4		84.3		7.8		89.4	
95.3		96.4		52.0		87.5	
94.5		93.6		71.0		0.0	
100		100		50.0		93.8	
95.4		82.0					
78.8		78.2					
95.4		94.7					
94.7		92.7					

**Table 3:** Viability of NTS of *S. mansoni* (percentage) at 72 h of exposure to anthelmintic drugs (PZQ, AS, and flubendazole (FBZ)) and anticancer drugs (vandetanib (VDT) and imatinib (IMT)) at 100 µM in vitro.

Viability (%)					
Vehicle Control (Mean)	Anthelmintic			Anticancer	
	PZQ (Mean)	AS (Mean)	FBZ	VDT	IMT
92.4	50.0	87.5	14.8	29.4	77.3
			0.0		72.3

**Table 4:** NTS viability (%) of NTS after exposure for 72 h to antioxidants alone at 100 µM, as assessed by staining with PI.

Viability (%)								
Control	NAC	Resv	Flav	Curc	Mel	OXA	Kaempf	DiPept
92.4	92.2	43.1	59.3	0.0	93.1	0.0	ND ^1^	88.9
(mean)		64.7	89.4		98.1			93.8
			67.6					

^1^ ND: not done.

**Table 5:** Viability of NTS (%) following exposure to PZQ, antioxidant and combination and its cooperative index.

	Viability (%)			Combination Index
	Anthelmintic	AntiOx	Combination	
PZQ+Resv	34.8	43.1	3.5	0.74
PZQ+Flav	7.8	59.3	72.9	4.90
PZQ+Curc	52.8	0.0	0.0	1.47
PZQ+Mel	52.8	93.1	31.4	0.80
PZQ+Kaempf	71.0	ND	ND	ND
PZQ+OXA	71.0	0.0	0.0	1.29
PZQ+DiPept	50.0	93.8	58.9	1.37
PZQ+NAC	50.0	92.2	59.3	1.42

ND: not done.

**Table 6:** Viability (%) and cooperative index for NTS exposed to AS, antioxidant and the combination.

	Viability (%)			Combination Index
	Anthelminthic	AntiOx	Combination	
AS+Resv	0.0	43.1	0.0	0.34
AS+Flav	89.4	59.3	49.2	1.01
AS+Curc	87.5	0.0	0.0	1.12
AS+Mel	87.5	93.1	95.7	4.51
AS+Kaempf	0.0	ND	ND	ND
AS+OXA	87.5	0.0	0.0	1.12
AS+DiPept	93.8	93.8	88.0	1.03
AS+NAC	93.8	92.2	84.3	0.89

ND: not done.

**Table 7:** Viability (%) of NTS following exposure to FBZ, antioxidant alone and the combination, and the cooperative index for each combination.

	Viability (%)			
	Anthelmintic	AntiOX	Combination	Combination Index
**FBZ + Resv**	0.0	64.7	0.0	1.35
**FBZ + Flav**	0.0	67.3	0.0	1.32
**FBZ + DiPetp**	14.8	93.8	95.7	21.25

**Table 8:** Viability of NTS (%) following exposure to anticancer drugs, antioxidants, drug combinations, and combination indexes.

	Viability (%)			
	Anticancer	AntiOx	Combination	Combination Index
**VDT + Flav**	29.4	67.6	0.02	1.03
**VDT + Resv**	29.4	64.7	0.09	1.06
**IMT + Flav**	77.3	89.4	77.1	1.45
**IMT + Resv**	72.3	64.7	64.1	1.75
**IMT + Mel**	77.3	98.1	91.3	2.83
